# Community assembly in mountain grasslands: decoupled aboveground versus belowground processes driven by contrasting soil resources

**DOI:** 10.3389/fpls.2026.1949546

**Published:** 2026-10-26

**Authors:** Qun Ma, Jianqiang Qian, Lu Zong, Zhimin Liu, Quanlai Zhou, Wei Liang, Jing Wu

**Affiliations:** 1 Institute of Applied Ecology, Chinese Academy of Sciences, Shenyang, Liaoning, China; 2 College of Forestry, Henan Agricultural University, Zhengzhou, Henan, China; 3 State Key Laboratory of Plant Diversity and Specialty Crops, Wuhan Botanical Garden, Chinese Academy of Sciences, Wuhan, China; 4 College of Life Science, Taizhou University, Taizhou, Zhejiang, China

**Keywords:** biodiversity conservation, bud bank, ecological restoration, functional traits, resource allocation, soil resource heterogeneity

## Abstract

**Introduction:**

Mountain habitats feature steep environmental gradients that shape specific vegetation diversity and soil heterogeneity. However, how plants coordinated aboveground and belowground reproductive strategies under varying resource availability remained poorly understood.

**Methods:**

We quantified the belowground bud bank, functional traits, and phylogenetic structures to elucidate plant resource allocation strategies governing community assembly in mountain grasslands of northern China. Six habitat types (SubAlpine Meadow, Forest-Edge Meadow, Floodplain Meadow, Hillside Steppe, Dry Steppe and Sandy Steppe) were selected based on resource availability along the elevation.

**Results:**

1) Subalpine Meadow exhibited the highest density and diversity. Floodplain Meadow and Dry Steppe had higher ramet density but lower diversity. Bud densities and diversities in the Subalpine, Floodplain and Hillside Steppe were higher than those in other low-elevation habitats; 2) None of the bud bank density, plant height and abundance showed strongly phylogenetic signals. The NTI, NRI were positively related with B/V density, while they were negatively correlated with height and B/V diversity indices; 3) Soil resources distinctly regulated aboveground and belowground populations: Total nitrogen, porosity and soil moisture were negatively correlated with diversities but positively with densities, acting as constraints on the density-diversity trade-off. Importantly, soil organic carbon specifically promoted belowground bud diversity, while total potassium drove aboveground ramet diversity.

**Conclusion:**

Our results revealed that deterministic processes (niche theory) governed community assembly in the Saihanwula mountain grassland. The aboveground and belowground populations in mountain grasslands were governed by distinct ecological processes and soil resources. Interspecific competition and environmental filtering drove aboveground community assembly, whereas resource availability constrained the belowground bud bank. Our findings provided significant insights for predicting the renewal and recovery potential of plants under global changes.

## 1 Introduction

Mountain environments host highly specialized flora and constitute hotspots of plant diversity (Myers et al., 2000; Wellstein and Kuss, 2011), and are characterized by steep environmental gradients (Di Musciano et al., 2018; Stanisci et al., 2011). High topographic diversity results in a high level of habitat diversity and community heterogeneity, and subsequently promotes great species richness (Rusch et al., 2011). Temperate mountain grasslands are characterized by harsh environmental conditions, including low temperatures, short growing seasons, and high solar radiation, which collectively impose strong abiotic filters on plant community assembly (Lin et al., 2025; Xiao et al., 2026). These ecosystems are also highly sensitive to ongoing climate change, making them ideal systems for investigating community assembly strategies under environmental stress (Li S. et al., 2026). Yet current studies on diversity patterns and the underlying drivers along elevations remain inconsistent across regions and scales, exhibiting monotonic declines, hump-shaped trends, or monotonic increases (Yue and Li, 2021; Zhang et al., 2021), or focusing on tropical, subtropical and temperate regions, with comparatively little attention paid to temperate mountain grasslands. Bridging biodiversity patterns is therefore essential for understanding distinctive ecological dynamics and community assembly of temperate mountain grasslands.

Many mechanisms have been proposed to explain why some collections of species coexist (Chase and Leibold, 2004; Hubbell, 2001; Vellend, 2010), including competition for limiting resources, and temporal fluctuations in environmental conditions and evolutionary processes (Spaak and Schreiber, 2023). Currently, community assembly mechanisms primarily rely on two theoretical frameworks: Niche theory attributes community assembly to deterministic processes (filtering and competition), whereas neutral theory invokes stochastic mechanisms (dispersal and drift). The former drives niche differentiation and coexistence (Tilman, 1982); the latter attributes community structure to random dynamics (Hubbell, 2001). However, current theories increasingly emphasize the integration of both processes rather than a single mechanism driving community assembly, giving rise to the modern coexistence theory (Chesson, 2000; HilleRisLambers et al., 2012). It provides a unifying framework for integrating these multiple processes: species coexistence depends on the balance between niche differentiation—which stabilizes coexistence by reducing interspecific competition—and mean fitness differences—which drive competitive exclusion. Within this framework, key ecological processes can be explicitly divided into biotic filters, abiotic filters and feedbacks between them. Understanding how these processes jointly regulate community assembly—particularly their relative contributions to niche differentiation versus mean fitness differences—is essential for predicting biodiversity responses to ongoing environmental change.

Interspecific competition directly contributes to fitness differences, as competitive ability differences among species determine which are more likely to exclude others; however, competition can also drive niche differentiation through character displacement (MacArthur and Levins, 1967). Resource utilization strategies—such as the differentiation in nutrient acquisition, water use, or carbon allocation—directly increase niche differentiation by allowing species to partition limiting resources (Tilman, 1982).

Plants fitness to different environments whether via colonization or shifts in local abundance— depend on both reproductive and extension traits (Hiiesalu et al., 2021; Tilman (2001)). These traits are predictors of plant performance (Kazakou et al., 2006), competitive ability (Grime et al., 1997), and stress tolerance (Wright and Westoby, 1999), and ultimately filter which species can establish in local habitats (Guisan and Rahbek, 2011; Gutiérrez-Girón and Gavilán, 2013). Aboveground ramets determined the spatial occupation and competitive ability of plant communities, while belowground bud banks served as a regenerative reservoir that buffered populations against stochastic disturbances and seasonal environmental fluctuations (Ma Q. et al., 2023). Despite the progress made in applying modern coexistence theory to community ecology, most empirical studies have focused on aboveground plant communities (Di Musciano et al., 2018), with comparatively little attention paid to the belowground module—particularly the belowground bud bank. In addition, the presence of a belowground bud bank can provide both a buffering effect and a supplementary mechanism after dispersal limitation has occurred (Klimešová and Herben, 2015). However, the role of the bud bank in modulating plant fitness and resource utilization ability—and thus its contribution to community coexistence—remains largely unexplored. Specifically, it is unclear whether aboveground and belowground plant communities are governed by the same ecological processes (e.g., environmental filtering, competition) or whether they respond differently to the same environmental gradients.

Abiotic factors in mountain environments play essential effects on species coexist and trait variability (Halassy et al., 2005; Wellstein and Kuss, 2011). Environmental filtering regulates the species pool that can potentially coexist, yet its effect on coexistence is twofold: moderate filtering may enhance niche differentiation by selecting functionally distinct species, whereas strong filtering may reduce niche differentiation by eliminating functionally diverse species (Kraft et al., 2015). Dispersal limitation acts as a stochastic filter that prevents potentially suitable species from arriving (Duan et al., 2025), whereas environmental filtering excludes species that arrive but cannot persist—together constituting two sequential filters in community assembly (Hubbell, 2001; Vellend, 2010). Among these factors, the soil resource availability plays an important role in vegetation distribution and population regeneration in mountain environments (Rusch et al., 2011; Sammul et al., 2003). Belowground bud bank vary with soil resource (Ma et al., 2019; Wu et al., 2020), while aboveground functional traits associated with resource acquisition strategies exhibit consistent shifts along soil gradients, being more conservative in resource-poor habitats and more dynamic in resource-rich environments (Rusch et al., 2011; Wellstein and Kuss, 2011). Therefore, it is essential for understanding how mountain plants integrate resource allocation strategies across both plant modules in response to environmental heterogeneity. Phylogenetic diversity and structure respond sensitively to environmental gradients, providing a robust proxy for tracking community succession and ecosystem function in mountain ecosystems (Zhang et al., 2023). Yet the evolutionary and ecological processes underlying these patterns remain poorly resolved (Culmsee and Leuschner, 2013; Wang et al., 2023). By integrating phylogenetic signals and jointly considering both phylogenetic and functional trait structures, it becomes possible to more accurately identify the key evolutionary and ecological processes that underpin species coexistence (Ma Z. J. et al., 2023).

To address these gaps, the present study was conducted in a temperate mountain grassland ecosystem along an elevation gradient. Specifically, we aimed to: (i) characterize the functional and phylogenetic diversity patterns of plant communities across an elevation gradient; (ii) test whether the observed community assembly processes aMace consistent with deterministic (e.g., environmental filtering) or stochastic (e.g., dispersal limitation) mechanisms; and (iii) determine how plants adjust their aboveground (ramet) and belowground (bud bank) modules in response to contrasting resource availability, thereby linking community-level patterns to species-level adaptive. We hypothesize that (H1) the belowground bud bank is primarily structured by environmental filtering, with soil resource heterogeneity driving trait-based assembly, whereas aboveground community is constrained by competitive exclusion; (H2) the decoupling between these two modules may represent a multi-modular coexistence mechanism, where the relative importance of niche-based versus neutral processes shifts along the elevation gradient: deterministic assembly dominates in resource-rich habitats, while stochastic processes dominate in resource-stressed habitats. strategies. This study will not only help us understand the assembly mechanism of plant communities in mountain grasslands, but also provide important theoretical basis for community resource allocation strategies in this area, and strengthen the comprehensive protection and utilization of mountain diversity in global changes.

## 2 Materials and methods

### 2.1 Study area

This study was conducted in the Saihanwula National Nature Reserve (E118°18′~118°55′, N43°59′~44°27′; **Figure 1**), Inner Mongolia of northern China, with a total area of 10.04×10^4^ ha, whichbelongs to the middle temperate–sub humid cold climate zone. It was an ecotone of agriculture and husbandry, that mainly protected the rare and endangered wild animals and plants, harboring a rich mosaic of forest, grassland, and wetland ecosystems. The area of meadows and steppes was 3.39×10^4^ ha, covering 33.7% of the total Reserve (Zhang, 2007). The community changed greatly and vegetation types were distributed vertically along the altitude, including subalpine meadow, dry steppe, hillside steppe, sandy steppe, floodplain meadow, forest-edge meadow and arbor forests. In this study, six communities along the altitude were selected, including Sandy Steppe (SA), Floodplain Meadow (FL), Dry Steppe (ST), Forest-Edge Meadow (FR), Hillside Steppe (HI), SubAlpine Meadow (AL) (**Supplementary Table S1**). The altitude of AL was generally above 1800 m, with a highest of 1997 m. The soil moisture in this zone was high due to the snow cover in winter and topographic rain in summer; The altitude of FR was located between 1200 and 1800 m, and this area was mainly dominated by trees and shrubs. The soil was fertile, and the habitat was superior. The ST and HI were at an altitude of 1100–1200m, with a rock fragment and poor water conditions. The SA was at an altitude of 1000–1100m, and characterized by sandy grass-steppe, with poor soil and water conditions. The FL was located in the valley areas, with superior moisture, soil and light conditions, and was dominated by wetland plants. The mean annual precipitation was 400mm, of which 70–80% fell during summer (June–August) and only 10–15% distributed across winter and spring (November–May in the following year). The mean annual temperature was 2°C. The annual low temperature in the coldest month (January) was -32°C, and the annual high temperature (AHT) in the hottest month (July) was 29°C.

**Figure 1:**
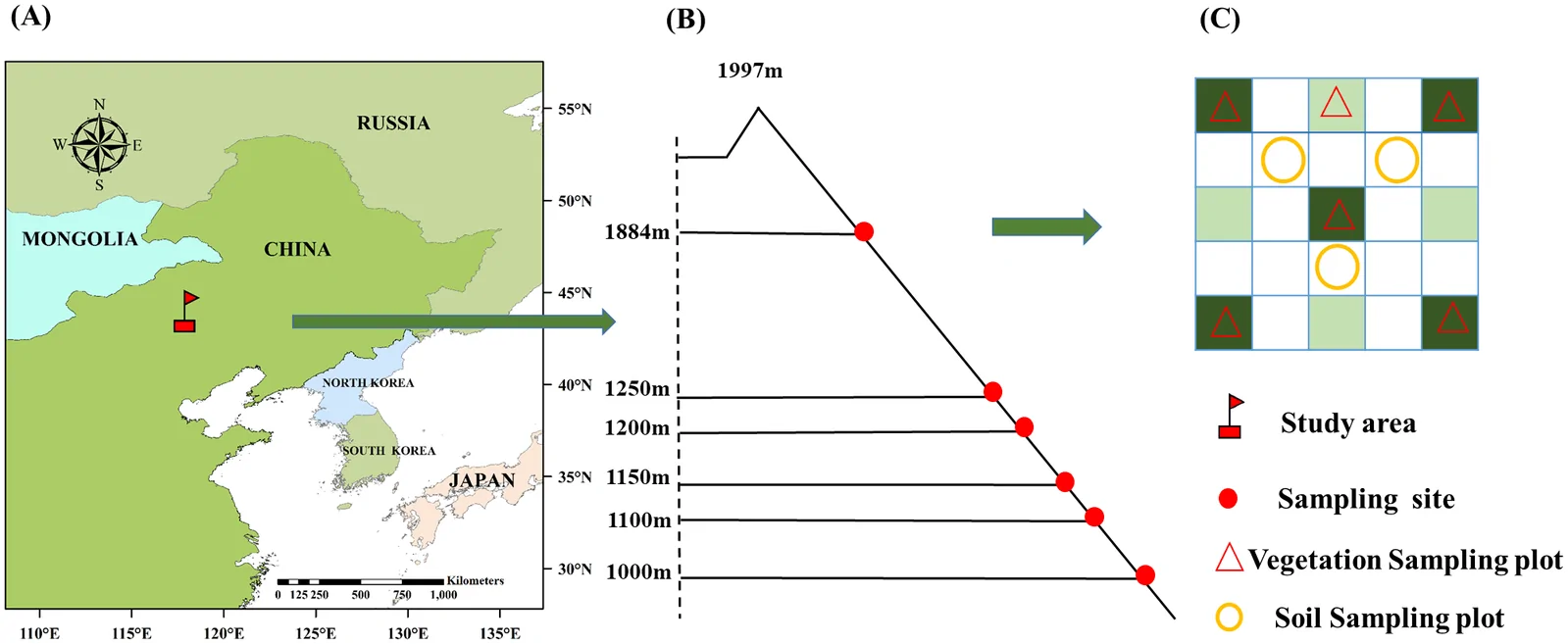
The geographical position of Saihanwula mountain in northeast China **(A)**, the location of the study sites with the respective dominant communities among different habitats **(B)**, the spatial pattern of six non-overlapping plots in a chessboard design **(C)**.

### 2.2 Sampling site

To minimize the potential effects of spatial heterogeneity, we preferentially selected sampling sites with relatively homogeneous environmental conditions. Along the elevation gradient, six habitats were selected and one site was selected at each habitat. At each site, six aboveground vegetation sampling plots (1m × 1m) (Zhang et al., 2026), six belowground bud bank (30 cm × 30 cm× 30 cm) plots and three soil sampling quadrats (100 cm × 50 cm) were selected (Tian et al., 2026; Qian et al., 2022). The 50-cm-deep soil pits were excavated solely to facilitate the collection of undisturbed soil cores using cutting rings at depths of 0–30 cm for measuring bulk density and soil particle size distribution. All soil samples used for chemical analyses were collected from the 0–30 cm depth layer. The spatial pattern of non-overlapping plots was based on a chessboard design (**Figure 1**).

#### 2.2.1 Aboveground plant community and belowground bud bank investigation

At each site, six aboveground vegetation sampling plots (1m × 1m) (Zhang et a., 2026) and six belowground bud bank (30 cm × 30 cm× 30 cm) plots were selected (Tian et al., 2026; Qian et al., 2022). The basic information was recorded for each quadrat, such as aboveground specie name, number, height, altitude etc., which were used for calculating the density and diversity indices. The connection between the aboveground ramets and belowground organs was kept intact for the subsequent identification of species and bud types. Soils adhering to the belowground parts were carefully removed, and bud bank samples were taken back to laboratory in plastic bags for bud identification and counting. The bud type and corresponding number in the quadrats were recorded separately. Only turgid bud tissues were counted, and necrotic or visibly dead tissues were discarded. Buds were categorized into eight types: RB (rhizome buds, formed mainly on the hypogeogenous and epigeogenous rhizomes), TRB (tiller and rhizome buds, formed mainly at the tiller bases and rhizomes), RSB (root-sprouting buds, adventitious buds formed mainly endogenously on roots), CB (collar buds, situated exogenously at the collar of the stem or shoot after soil burial) and BB (bulb buds, in the bulbs).DB (dicotyledonous bud, buds at the dicotyledonous rhizomes), TB (Tuber bud), and SB (Stolon bud). Buds on rhizomes and roots could be counted directly, whereas leaf tissues, ramets, or bulb bases required dissection for the counting of tiller and bulb buds.

#### 2.2.2 Soil samplings and analysis

At each site, three quadrats (100 cm × 50 cm) were selected for soil sampling. The soil pits were excavated to the depth of 50 cm to collect samples for soil bulk density and porosity at the depth of 0–30 cm, using a standard container, with 100 cm^3^ in volume (50.46 mm in diameter and 50 mm in height). Six soil cores (8 cm in diameter) were taken in each quadrat randomly and these cores were composited into a single sample, at depths of 0–30 cm for measuring soil water content (SWC), pH, total nitrogen (TN), total phosphorus (TP), total potassium (TK), available P, available N, available K and soil organic carbon (SOC; Ma et al., 2017). The pH was measured in a water-filtrate suspension with a mass-to-volume ratio of 1:2.5 (Rayment and Lyons, 2011; van Lierop, 1981). The SOC was determined by oxidation with potassium dichromate and sulphuric acid (Walkley and Black, 1934). TN was determined by the Kjeldahl method (Bremner, 1996). TP was were measured by ICP (Zhang et al., 2025). The available P was determined using the Olsen procedure (Olsen et al., 1954) and soil porosity using granulometric analysis of five fractions without decarbonation (Flint and Flint, 2002).

### 2.3 Phylogeny and community phylogenetic structure

The phylogeny tree of species in this study area was constructed under the APG III framework. Every recorded species was first standardized and validated against The Plant List (TPL), using an archived version of the TPL website via the Internet Archive Wayback Machine, and cross-checked against the World Flora Online (http://about.worldfloraonline.org) database. The tree was then generated with the package V.PhyloMaker 2 in R 4.1.2 and subsequently visualized and annotated with ggtree. The phylogeny was composed of nodes (representing taxonomic units) and branches (depicting evolutionary relationships); branch lengths quantified the estimated magnitude of divergence along the underlying phylogenetic history. According to the data, this study involved a total of 69 species and 7 bud bank types (**Figure 2**).

**Figure 2:**
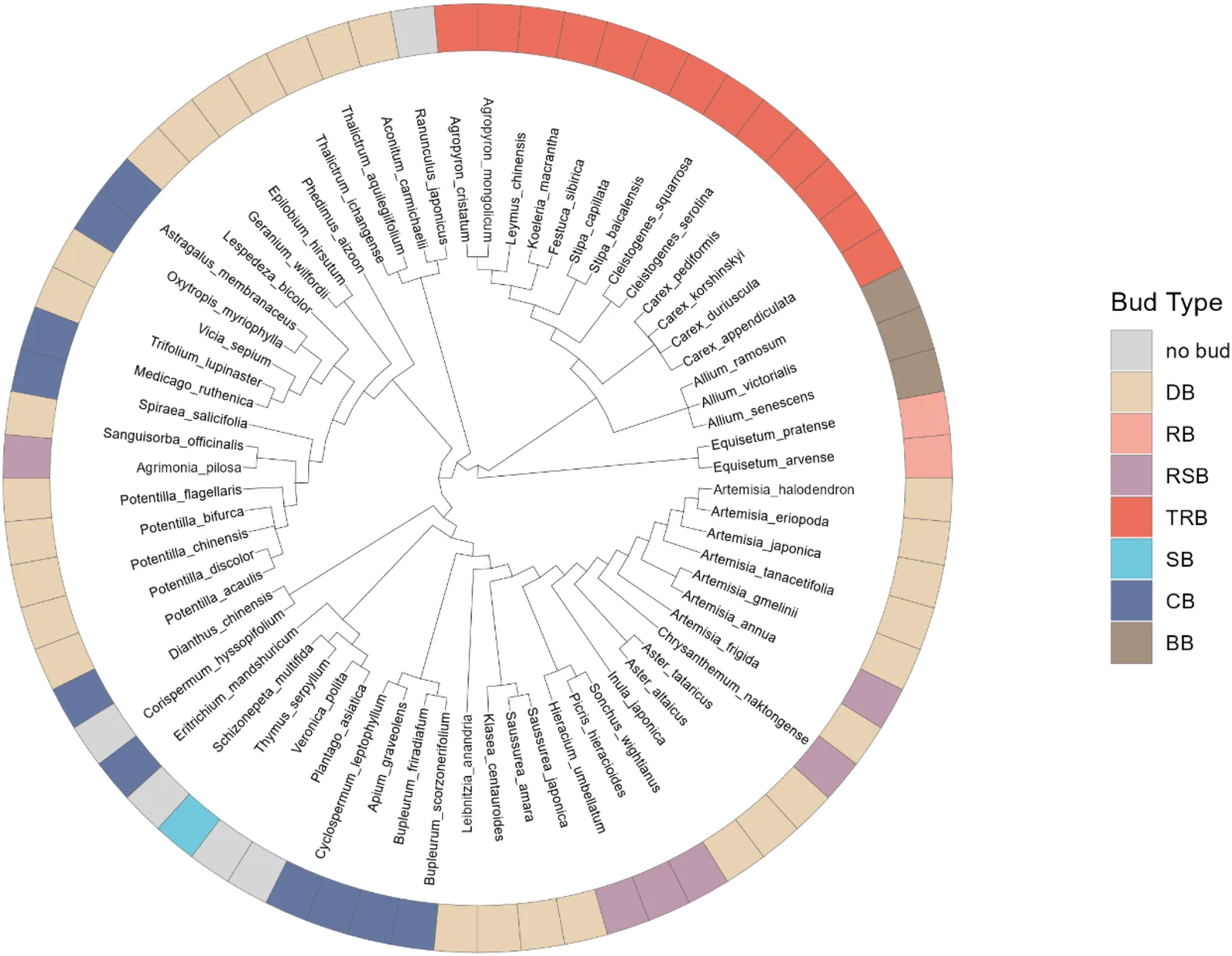
Phylogenies of species and bud type in mountain communities in the Saihanwula Mountain of northern China. RB (rhizome bud), TRB (tiller and rhizome buds), RSB (root-sprouting buds), CB (collar buds), BB (bulb buds), DB (dicotyledonous rhizome bud), TB (Tuber bud), SB (Stolon bud).

The phylogenetic diversity (PD) was used to quantify phylogenetic diversity. It represented the total length of the evolutionary branches of the community species on the phylogenetic tree. The net relatedness index (NRI) and the nearest taxon index (NTI) were used to determine the phylogenetic structure of the community. The NRI calculated the mean phylogenetic distance (MPD) among all species in the community, while the NTI calculates the mean distance between any species in the community and its nearest related species (Mean Nearest Taxon Distance, MNTD). NRI emphasized the similarity among species, while NTI focuses on the influence exerted by similar species. The NTI and NRI of each sample site were calculated using the “picante” package in R 4.1.2. If NRI > 0 and NTI > 0, it indicated that the phylogenetic structure of the community was convergence, and habitat filtering played a dominant role, assembling a community of closely related species that shared functionally similar traits; if NRI < 0 and NTI < 0, it indicated that the phylogenetic structure of the community tended to be divergent, and The species exhibited comparatively distant relationship, with interspecific competition and trait-similarity constraints serving as key drivers of community assembly; if NRI = 0 and NTI = 0, it indicated that the phylogenetic structure of the community was stochastic, with no obvious trend of kinship convergence or divergence (Webb, 2000).

### 2.4 Phylogenetic signal of the functional traits

The phylogenetic conservatism of functional traits was assessed using the K statistic by the Brownian motion evolutionary model (Blomberg et al., 2003). The K was computed using the “ape” and “picante” packages in R 4.1.2. These values were evaluated against a null model generated through 999 random permutations. A significantly higher observed K value compared to the null distribution indicated the presence of a significant phylogenetic signal (*p* < 0.05). When K = 1, functional traits evolved independently of phylogenetic structure, consistent with the expectations of Brownian motion and implying a random evolutionary pattern; If K < 1, the phylogenetic signal was weaker than random model, suggesting convergent evolution in functional traits across species; Conversely, if K > 1, the phylogenetic signal was stronger than expected under randomness, indicating that functional traits were phylogenetically conserved and exhibited greater similarity among closely related species.

### 2.5 Data statistics

We used a stepwise analytical framework to disentangle potential drivers of community assembly. We first tested whether aboveground and belowground community structures differed significantly among habitats. After confirming significant compositional differences among habitats, we conducted phylogenetic signal tests for key traits to rule out phylogenetic constraints. We then performed RDA to identify the environmental variables most strongly associated with community variation, and interpreted the assembly mechanisms based on habitat-specific environmental characteristics.

All data were processed and analyzed using Microsoft Excel 2016. FD and PD were calculated using the “picante” packages in R 4.1.2. The heatmap was plotted using the “ pheatmap “ packages in R 4.1.2. One-way analysis of variance (ANOVA) was performed to assess significant differences in density, diversity indices, and PD across different altitudinal gradients. Multiple comparisons among different habitats were performed using Fisher’s Least Significant Difference (LSD) test to determine the significance of differences between groups (*p* < 0.05). Additionally, redundancy analysis (RDA) was conducted using the “vegan” package in R4.1.2 to examine the relationships between soil environmental factors and diversity indices. The RDA model in our study included a comprehensive set of 12 environmental variables: SWC, pH, total N, total P, total K, available N, available P, available K, bulk density, porosity, SOC and elevation. Environmental variables were selected via forward selection in RDA, and only those with a significant contribution (*p* < 0.05) were retained in the final model. The box plots and bar charts were plotting in Origin 2021.

## 3 Results

### 3.1 The aboveground ramet density, height and diversity among all habitats

The ramet densities of AL, FL and ST were 2555.56 No.m^-2^, 2616.67 No.m^-2^, 1894.44 No.m^-2^respectively, which were significantly higher than those of the other habitats (**Figure 3A**). Conversely, the diversity indices of AL, FR and SA were significantly higher than those of other three habitats (*p* < 0.05, **Figure 3C**). The heights of SA and FL (13.36cm, 6.80cm) were significantly lower than the other habitats (*p* < 0.05, **Figure 3B**).

**Figure 3:**
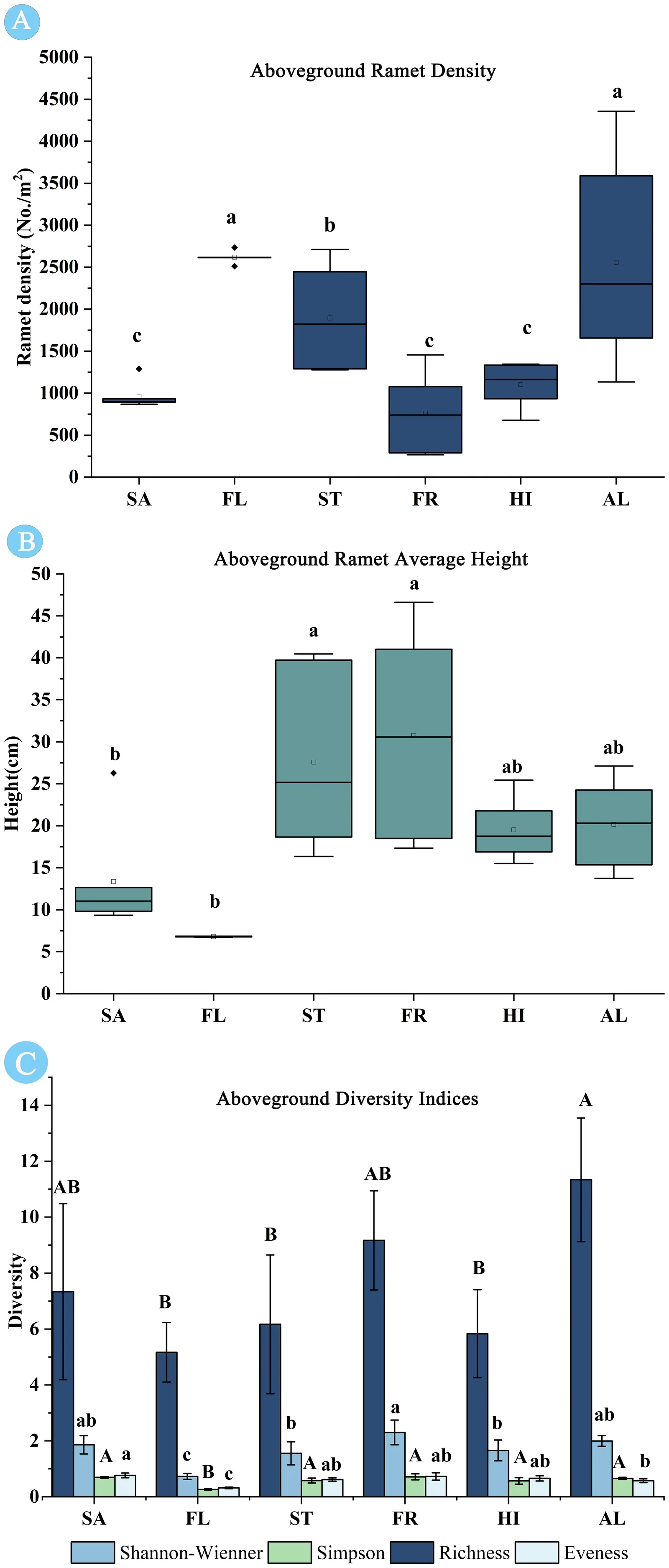
The vegetation height, ramet density and diversity among all habitats. **(A)** was the plant ramet density, **(B)** was the vegetation height and **(C)** was the ramet diversity indices. Sandy Steppe (SA), Dry Steppe (ST), Hillside Steppe (HI), Forest-Edge Meadow (FR), Floodplain Meadow (FL), SubAlpine Meadow (AL). “abc”/”ABC” represented the significance test. Different letters indicated the density/height/diversity showed significant differences among different habitats (*p* < 0.05), while the same letter indicated no significant differences.

### 3.2 The belowground bud density and diversity among all habitats

The belowground bud density increased significantly with the increase of elevation, except for those in floodplain. The total and tiller bud densities in the FL, AL and HI were 2784.21 No.m^-2^, 2151.85 No.m^-2^and 1287.04 No.m^-2^ respectively, which were significantly higher than those in other habitats. Rhizome bud densities in the FL was 784.18 No.m^-2^, the highest among all habitats. Root-sprouting bud densities in the FR and AL were 329.63 No.m^-2^and 261.11 No.m^-2^, higher than those in the other habitats, which was rarely observed in the FL. The other bud types were also found in HI and AL (**Figures 4A–E**). Similarly, the bud diversity indices of FL, AL and HI were significantly higher than those in other habitats. No significant differences in bud Simpson diversity were detected among habitats (**Figure 4F**).

**Figure 4:**
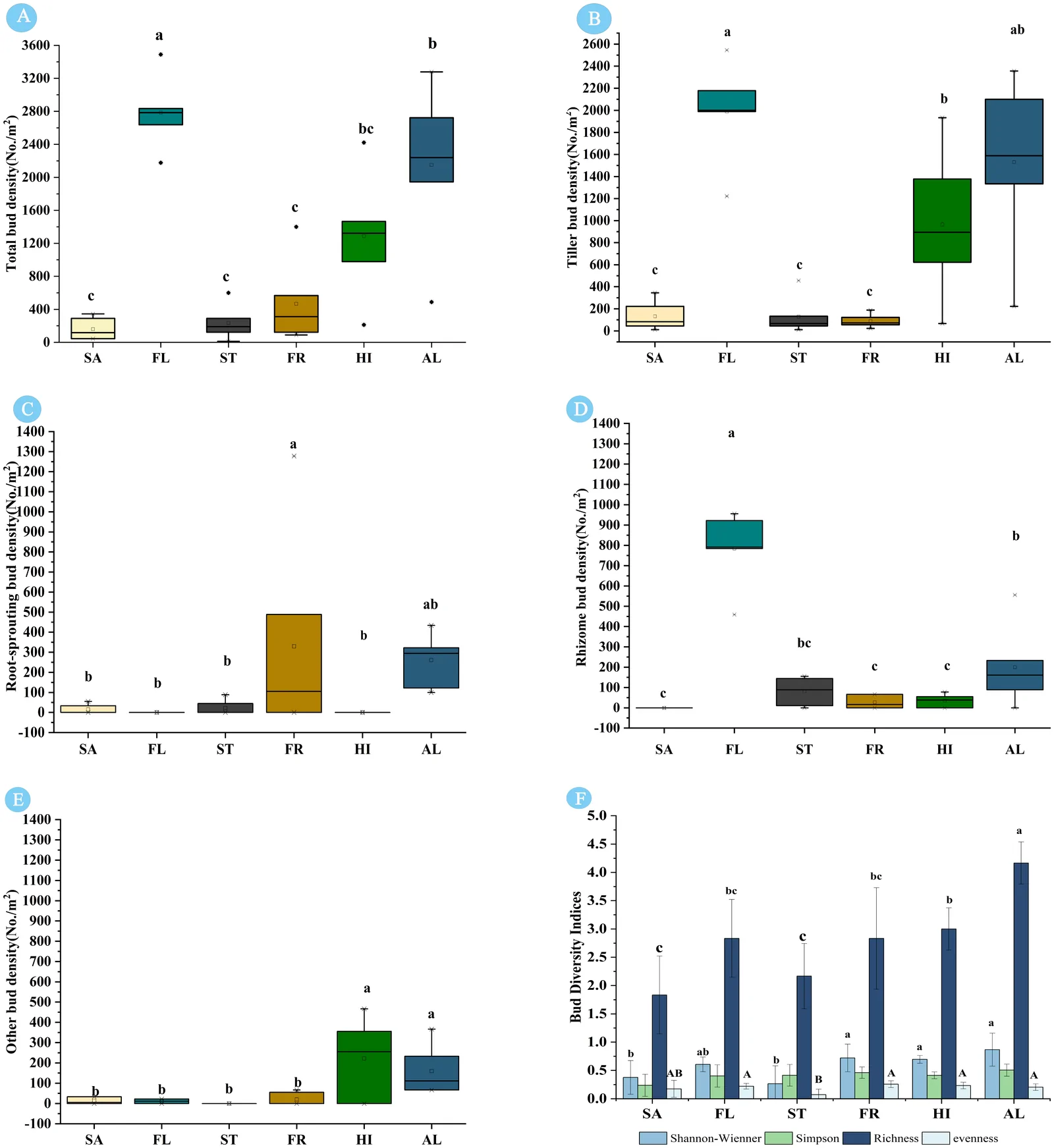
The bud bank densities and diversity among all habitats. **(A–E)** were the total bud density, tiller bud density, root-sprouting bud density, rhizome bud density and other bud density, respectively. **(F)** was the bud diversity indices. Sandy Steppe (SA), Dry Steppe (ST), Hillside Steppe (HI), Forest-Edge Meadow (FR), Floodplain Meadow (FL), SubAlpine Meadow (AL). “abc” represented the significance test. Different letters indicated the density showed significant differences among different habitats (*p* < 0.05), while the same letter indicated no significant differences.

### 3.3 The phylogenetic conservation, structure and relationships with aboveground vegetation and belowground bud bank

The height, ramet richness and bud density showed no significant phylogenetic signals (K<1; *p*> 0.05), indicating these functional traits were largely shaped by contemporary environmental filtering and biotic interactions rather than by phylogenetic constraints (**Table 1**). The NRI, NTI of all habitat were <0 (p>0.05, except for FL), indicating that the phylogenetic structure of the community did not deviate significantly from random expectation, and that the deterministic signals of environmental filtering and competitive exclusion were weak. The PD didn’t change significantly among different habitats (**Table 2**), and showed positive relationships with vegetation and bud richness (**Figure 5**). Bud density was positively related with vegetation density. The NTI, NRI were positively related with B/V density, while they were negatively correlated with height and B/V diversity indices (Evenness, Simpson and Shannon-wiener diversity).

**Table 1:** The phylogenetic signal of plants functional traits in communities.

Functional traits	K	*p*
Richness	0.0027	0.725
Height	0.0205	0.177
Bud density	0.0181	0.220

**Table 2:** The phylogenetic structure and diversity among different habitats.

HABITATS	PD	NRI	*P*_NRI_	NTI	*P*_NTI_
SA	767.79	-0.66	0.307	-1.01	0.170
FL	748.37	3.164	0.994	1.51	0.912
ST	799.91	-1.103	0.071	-0.80	0.235
FR	811.69	-1.216	0.077	-1.23	0.120
HI	751.11	-0.714	0.230	-2.06	0.010
AL	837.31	-0.59	0.478	-0.96	0.190

**Figure 5:**
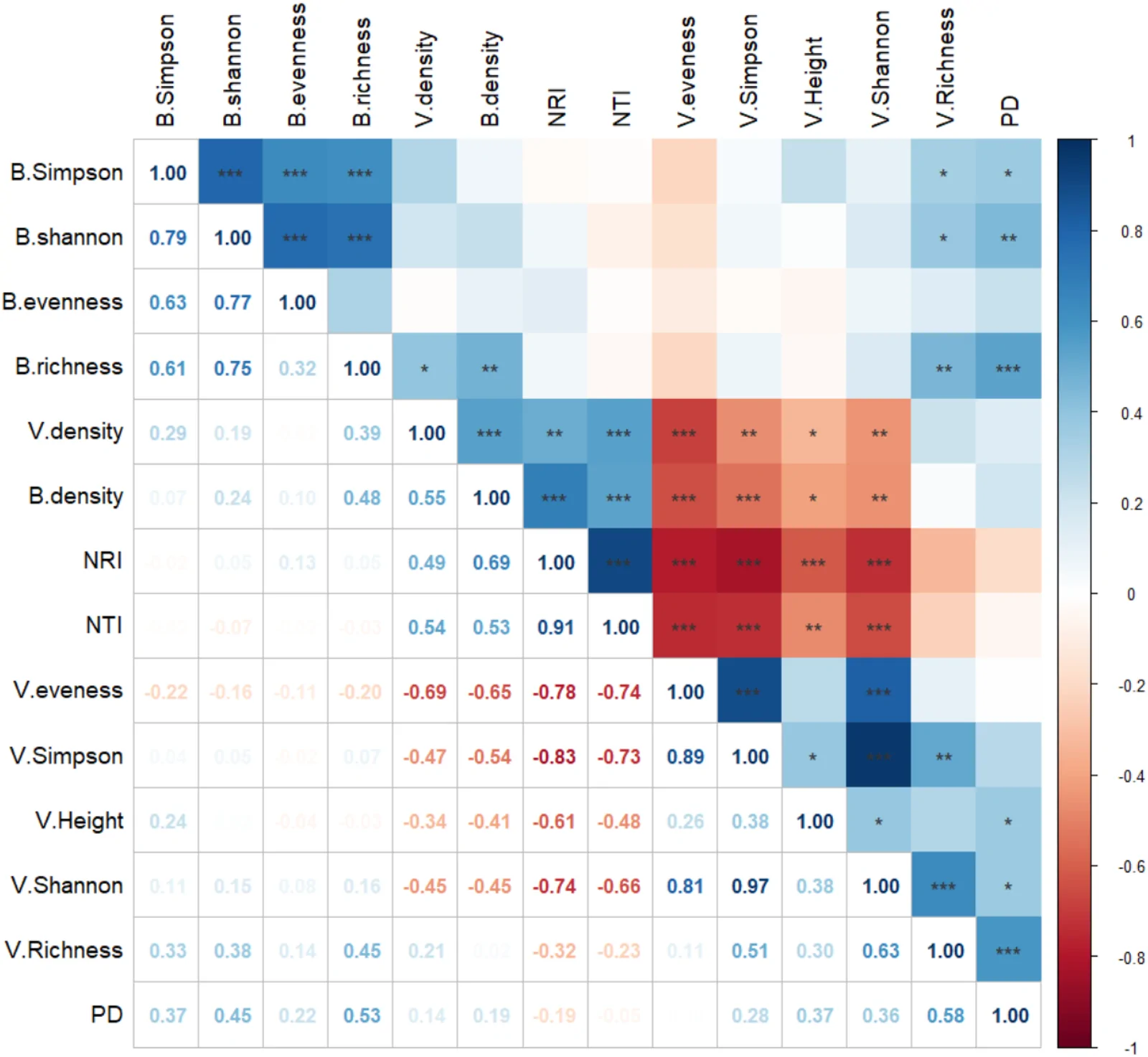
The autocorrelation between vegetation, bud bank and phylogenetic structure. “B.” represented Bud and “V.” represented Vegetation. Blue indicated positive correlations, while red indicated negative correlations. Darker shades represented stronger correlations.

### 3.4 The influences of resource availability on bud bank and vegetation

The contributions of soil factors on bud variation and vegetation variation were 53.3% and 67.5%, respectively (**Table 3**). The dominant contributor for bud density was TN and that for bud diversity was SOC. The dominant contributor for aboveground vegetation density was TN, SWC and porosity, that for aboveground vegetation diversity was TK. The TN, porosity and SWC were negatively related with vegetation and bud diversity indices, while positively with vegetation density and bud densities (**Figure 6**). The SOC was positively related with bud diversity indices and TK was positively related with vegetation diversity indices.

**Table 3:** The soil factors’ contributions on bud and vegetation variations.

Vegetation modules	Soil factors	Explains (%)	Contribution (%)	*p*-Value
Belowground Bud Bank	TN	22.7	42.6	0.002**
	SOC	17.1	32.0	0.002**
	SWC	5.4	10.2	0.022**
	Porosity	5.0	9.3	0.026*
	TK	3.1	5.9	0.076
	Total	53.3		
Aboveground Vegetation	TN	27.8	41.2	0.002**
	TK	14.0	20.7	0.002**
	Porosity	12.3	18.3	0.002**
	SWC	10.3	15.3	0.002**
	SOC	3.1	4.6	0.05
	Total	67.5		

SOC, Soil Organic Carbon; SWC, Soil Water Content; TK, Total Kalium; TN, Total Nitrogen. *p < 0.05; **p < 0.01.

**Figure 6:**
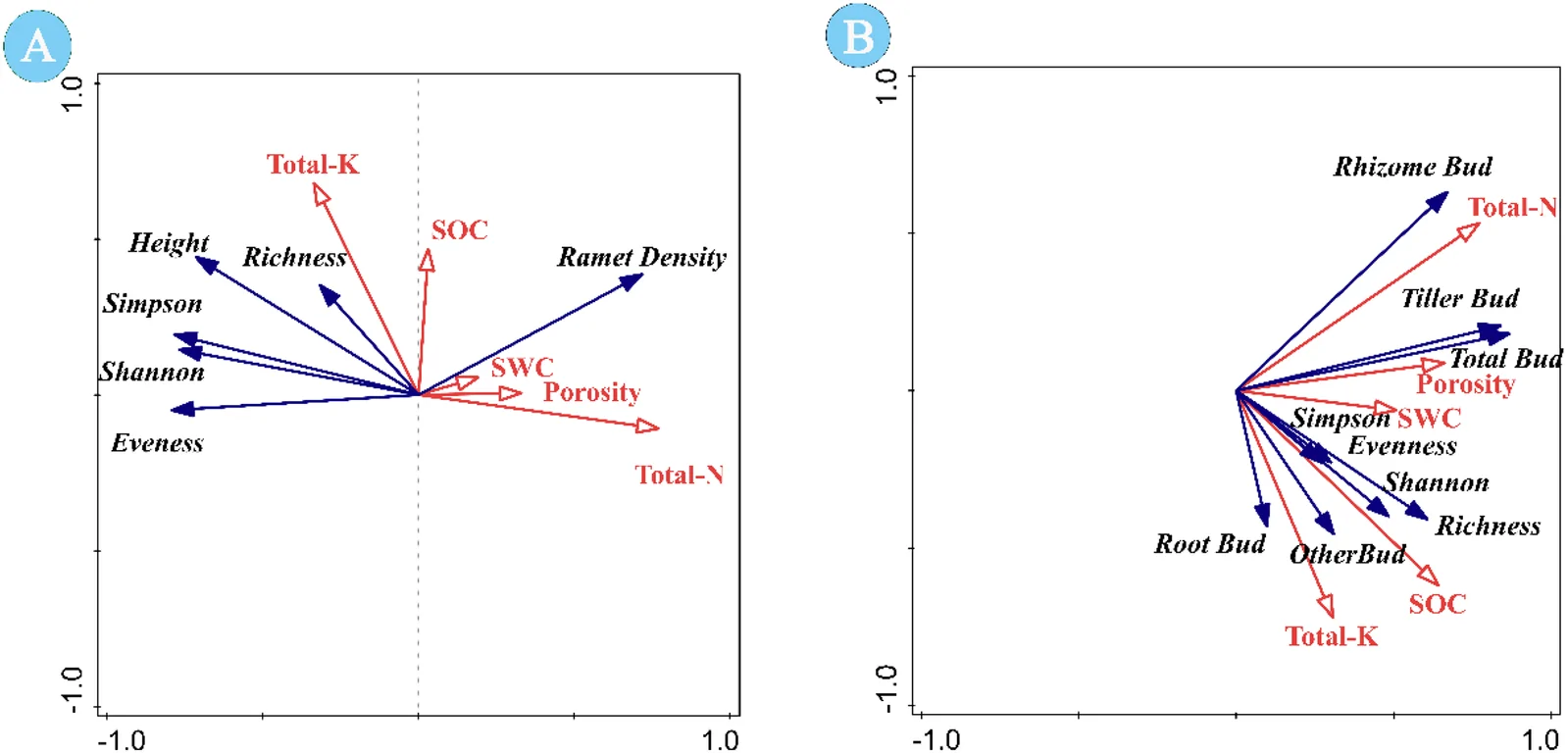
The influences of soil factors on vegetation **(A)** and bud bank **(B)**. Red arrows represented soil factors, while blue arrows represented aboveground ramet or belowground bud bank traits.

## 4 Discussion

The dynamics of the belowground bud banks reflected plant adaptation to environmental changes, served as a reliable proxy for forecasting community structure and ecosystem function (Luo et al., 2023). Herbaceous plants were known to be highly sensitive to local environmental conditions (Evette et al., 2009; Zhang et al., 2016), and were more strongly shaped by micro-habitats than by broad-scale gradients. Consistent with earlier reports of weak altitudinal trends in herb richness (Bhattarai and Vetaas, 2003; Desalegn and Beierkuhnlein, 2010; Ye et al., 2020), we found that aboveground ramet density and diversity showed no significant changes along the elevation gradient. In contrast, belowground bud bank density and diversity exhibited an increasing trend with elevation. In this study, tiller and rhizome buds predominated and their positive correlations with aboveground density suggested that the grassland productivity was associated with the regenerative capacity of the belowground bud bank, consistent with the view that the bud bank acts as a critical source of population renewal in mountain grasslands (Hiiesalu et al., 2021), thereby providing an energy reserve that enabled rapid regeneration once conditions ameliorate (Ma Q. et al., 2023). The strikingly higher bud densities and diversity in the AL, FL, and HI meadows suggested that habitat-specific resource availability exerted a strong filter on belowground regenerative strategies. In resource-poor habitats, we observed a dominance of resource-conservative species (those relying on root-sprouting and tiller bud banks, Ma et al., 2019), whereas resource-rich patches were characterized by resource-acquisitive species (e.g., rhizomatous clonal plants). This functional differentiation in resource use was a more effective predictor of community structure than phylogenetic relatedness, supporting the view that contemporary ecological processes, such as environmental filtering and niche partitioning, often override evolutionary constraints in community assembly (Halbritter et al., 2025).

The Habitat-filtering hypothesis posits that only species whose functional traits match the abiotic constraints of a given site can establish and persist there. In cold, high-elevation systems the range of viable trait combinations was narrow, strengthening phylogenetic conservatism (Yue and Li, 2021). Environmental filtering increased trait similarity among co-occurring taxa and assembled communities dominated by closely related, convergent adapted species. In this study, NRI and NTI were significantly positively correlated with community density but negatively correlated with species diversity. These correlations suggested that phylogenetic clustering (high NRI/NTI) arose when environmental filters select for closely related, clonal dominants that achieved high densities through bud bank proliferation, while competitively excluding other species (low diversity). Conversely, phylogenetic overdispersion (low NRI/NTI) was associated with high diversity, where niche differentiation among distantly related species allowed coexistence without competitive exclusion. These findings indicated that density and diversity were not merely demographic descriptors, but were systematically linked to the underlying phylogenetic structure of the community, reflecting the opposing forces of environmental filtering (promoting density) and competitive exclusion (promoting diversity) across habitats. Interestingly, FL produced high above-ground ramet density and a large below-ground bud bank, yet generated low species richness, high evenness, and limited niche differentiation, indicating efficient but taxonomically narrow utilization. On the contrary, the niche-differentiation hypothesis stated that interspecific competition intensifies with trait similarity; species must diverge in niche space to avoid competitive exclusion. The more closely related and functionally alike two taxa are, the greater the overlap Vegetation modulesin resource requirements and the lower the probability of stable coexistence (Catford et al., 2012; Lambdon et al., 2008). This theory suggested trait divergence among co-occurring plants: intense competition for identical resources drove competitive exclusion of closely related, functionally similar taxa. Consequently, only species that were phylogenetically and ecologically distinct could persist together, yielding a community structured by phylogenetic over-dispersion. In this study, across all habitats except FL, interspecific competitions may be intensified, resources were fully explored, and species may expand into broader niche space, allowing competitive exclusion to dominate community assembly. Aboveground diversity and density were negatively correlated, indicating that conspecific negative density also shaped the aboveground community in these habitats. Additionally, the positive correlation of PD and species richness was due to that the higher the species richness, the more species characteristics were overlapped, and lengthened the community’s phylogenetic branch length (Yang et al., 2004), which were consistent with Lv et al. (2023). Previous studies have documented a shift from phylogenetic divergence to convergence with increasing elevation in alpine ecosystems (2000-5000m; Huang et al., 2010). In contrast, our study did not detect significant changes in phylogenetic structure across habitats, which may be attributable to the lower elevation range of our study area (1000-1900m). Soil properties in mountain grasslands vary with elevation and were closely linked to community distribution and plant adaptive strategies. Previous studies have identified SWC, available P, TK, and TN as key factors linked to alpine meadow diversity (Li et al., 2019; Lv et al., 2023). Our study found soil factors exhibited a functional differentiation. This pattern suggested a dual role of soil resources: ‘promoting resources’ (TN, SWC, porosity) appeared to enhance density but suppress diversity, likely via competition, whereas ‘constraining resources’ (TK, SOC) may support diversity through belowground regeneration. Thus, soil resource heterogeneity may mediate the productivity–diversity trade-off. Environmental stress may intensify aboveground competition and rapidly eliminate rare species. However, an abundant belowground bud bank—especially under favorable SOC and TK conditions—may buffer such diversity losses (Zhang et al., 2024). The FL meadow, with higher SWC, SOM and N/P availability, illustrated this pattern, maintaininghigh densities of both aboveground ramets and belowground bud banks (rhizome and tiller buds). With light and nutrients equally accessible, vertical competition relaxed and the weighted average height of the community declined (Liira and Zobel, 2000). In the FR meadow, moderate nutrients combined with lateral shading likely promoted height growth at the expense of lateral expansion, resulting in a taller community but lower ramets and bud densities. Meanwhile the bud bank shifted toward root-sprouting buds -a disturbance-tolerant strategy we have previously documented (Ma et al., 2019). The AL meadow at 1900m exhibited the highest density and diversity (both aboveground and belowground parts) among all habitat types. This pattern likely resulted from the combination of abundant soil moisture, high nutrient availability and the highest TK, supporting plant root extension, enhanced nutrient-use efficiency and sustained high photosynthetic rates, translating into larger aboveground biomass (Yuan et al., 2019). The higher bud bank diversity at high elevation and in low-resource conditions suggested that environmental filtering may not be overly restrictive; instead, it may enhance niche differentiation via the bud bank. At SA, ST, and HI steppes, prolonged overgrazing and trampling may have induced soil compaction, accompanied by declines in SWC, porosity and permeability (Zhang et al., 2018). This may have limited root growth and nutrient availability, potentially leading to declines in both ramet and bud bank densities. The vegetation productivity and soil chemical properties interacted with each other to re-change the soil environment and completing a negative plant–soil feedback that eroded species diversity across these habitats (Ma et al., 2020; Shao et al., 2022). Although aboveground communities are structured by competition, the bud bank may mitigate the resulting mean fitness differences by providing a buffer for weaker competitors. Habitat-specific variation in bud bank density likely reflected differences in plant resource-use strategies.

## 5 Conclusion

In this study, deterministic processes (niche theory) governed community assembly in the Saihanwula mountain grasslands, with their effects reversing along the elevation gradient. Aboveground communities were primarily influenced by interspecific competition and environmental filtering, whereas belowground bud bank was positively regulated by soil resources availability. The density–diversity trade-off was jointly constrained by TN, SWC and porosity. Environmental filtering and biotic interactions may exert stronger selective pressure on traits than did phylogenetic signals in the mountain communities. This study provided novel insights into mountain community assembly by demonstrating that aboveground and belowground modules were governed by fundamentally different assembly rules, and highlights the critical role of soil resource heterogeneity in mediating this decoupling, offering a framework for predicting vegetation responses to ongoing environmental change in mountain ecosystems. Despite these novel insights, several limitations should be acknowledged. Our assessment was limited to plant abundance and bud bank functional traits; we did not include key leaf, root economic traits (e.g., specific leaf area, leaf nitrogen content, root diameter, and tissue density), or microbial diversity. Future studies will incorporate a broader suite of functional traits to provide a more comprehensive mechanistic understanding of community assembly in temperate ecosystems. Another potential limitation of this study was that sampling was confined to a single nature reserve with only one meteorological station, which prevented us from capturing climatic heterogeneity (e.g., precipitation and temperature) across different habitats. Future studies incorporating high-resolution climatic data or multi-site observations are needed to further validate the generality of our findings. Moreover, future research will integrate global-scale forest ecosystem datasets to enable a more generalizable assessment of community assembly mechanisms across diverse biomes.

## Data Availability

The raw data supporting the conclusions of this article will be made available by the authors, without undue reservation.

## Supplementary material

Supplementary Table 1 The habitat types among different habitats in Saihanwula Reserve.
