# Supplementary material for: Community assembly in mountain grasslands: decoupled aboveground versus belowground processes driven by contrasting soil resources
Source: Front Plant Sci. 2026 Oct 26;17:1949546. doi: 10.3389/fpls.2026.1949546 (PMC13745469; doi:10.3389/fpls.2026.1949546)
Supplement: Supplementary file 1 [file Table1.docx]

**Table S1** The different habitat types in Saihanwula Reserve

| elevation  (m) | Habitat | Community | SWC  (%) | SOC  (g/kg) | TN  (g/kg) | TP  (g/kg) | TK  (g/kg) | Porosity  (%) | pH |
| --- | --- | --- | --- | --- | --- | --- | --- | --- | --- |
| 1884 | Subalpine meadow | Dominant species: *Leucopoa albida*  Companion species: *Vicia unijuga*、*Sanguisorba officinalis L.* 、*Aster tataricus*、*Carex duriuscula*、*Artemisia tanacetifolia* | 49.44 | 39.29 | 3.64 | 1.49 | 52.98 | 47 | 5.70 |
| 1259 | Hillside steppe | Dominant species: Filifolium sibiricum  Companion species: *Bupleurum scorzonerifolium*、*Leymus chinensis*、*Stipa baicalensis*、*Artemisia gmelinii*、*Artemisia tanacetifolia*、*Medicago ruthenica*、*Carex pediformis* | 20.74 | 44.90 | 3.59 | 2.02 | 51.67 | 59 | 6.03 |
| 1200 | Forest-edge meadow | Dominant species: *Sanguisorba officinalis*  Companion species: *Carex pediformis*、*Artemisia tanacetifolia*、*Artemisia gmelinii* | 42.13 | 56.87 | 3.20 | 3.90 | 46.15 | 54 | 5.76 |
| 1145 | Dry steppe | Dominant species: *Stipa baicalensis*  Companion species: *Cleistogenes squarrosa*、*Leymus chinensis*、*Filifolium sibiricum*、*Artemisia tanacetifolia*、*Festuca dahurica* | 20.74 | 18.75 | 2.89 | 1.34 | 42.28 | 47 | 6.50 |
| 1100 | Floodplain meadow | Dominant species: *Carex appendiculata*  Companion species: *Potentilla anserina*、*Sanguisorba officinalis*、*Agrostis clavata Trin.*、*Ligularia sagitta*、*Inula japonica Thunb.* | 43.00 | 65.00 | 8.02 | 14.00 | 26.00 | 65 | 6.82 |
| 1000 | Sandy steppe | Dominant species：*Agropyron mongolicum*  Companion species：*Setaria viridis*、*Corispermum hyssopifolium L.*、*Agropyron cristatum*、*Carex duriuscula* | 37.53 | 9.04 | 1.60 | 3.40 | 12.5 | 30 | 6.80 |
